# Quantitative Imaging of Young's Modulus of Soft Tissues from Ultrasound Water Jet Indentation: A Finite Element Study

**DOI:** 10.1155/2012/979847

**Published:** 2012-08-15

**Authors:** Min-Hua Lu, Rui Mao, Yin Lu, Zheng Liu, Tian-Fu Wang, Si-Ping Chen

**Affiliations:** ^1^National-Regional Engineering Laboratory for Key Technology of Medical Ultrasound, Shenzhen University, Shenzhen 518060, China; ^2^Guangdong Key Laboratory of Biomedical Information Detection and Ultrasound Imaging, Shenzhen University, Shenzhen 518060, China; ^3^Department of Biomedical Engineering, School of Medicine, Shenzhen University, Shenzhen 518060, China; ^4^Shenzhen Key Labratory of Service Computing and Applications, College of Computer Science and Software Engineering, Shenzhen University, Shenzhen 518060, China

## Abstract

Indentation testing is a widely used approach to evaluate mechanical characteristics of soft tissues quantitatively. Young's modulus of soft tissue can be calculated from the force-deformation data with known tissue thickness and Poisson's ratio using Hayes' equation. Our group previously developed a noncontact indentation system using a water jet as a soft indenter as well as the coupling medium for the propagation of high-frequency ultrasound. The novel system has shown its ability to detect the early degeneration of articular cartilage. However, there is still lack of a quantitative method to extract the intrinsic mechanical properties of soft tissue from water jet indentation. The purpose of this study is to investigate the relationship between the loading-unloading curves and the mechanical properties of soft tissues to provide an imaging technique of tissue mechanical properties. A 3D finite element model of water jet indentation was developed with consideration of finite deformation effect. An improved Hayes' equation has been derived by introducing a new scaling factor which is dependent on Poisson's ratios *v*, aspect ratio *a/h* (the radius of the indenter/the thickness of the test tissue), and deformation ratio *d/h*. With this model, the Young's modulus of soft tissue can be quantitatively evaluated and imaged with the error no more than 2%.

## 1. Introduction

Physiologic processes may change tissue properties significantly. Tissue elasticity is one of the typical mechanical properties which generally correlate with pathological changes [1], such as cancer [2] and osteoarthritis [3]. During the past a few decades, there have been many efforts to develop various techniques to measure or image the elasticity of soft tissue, such as elastography [4–6], indentation [7–9], and atomic force elasticity microscopy [10–12].

Indentation is one of the most commonly used method to measure mechanical properties of soft tissues *in situ* or *in vivo*, because it does not require special preparation of regular shaped tissues and can be employed to perform tests on small specimens [13]. A typical application of indentation is to assess the degeneration of articular cartilage (AC). AC is often characterized as a single phase, isotropic, homogeneous linear elastic model when no interstitial fluid flows during instantaneous, and equilibrium responses [7, 8]. Rigid cylindrical flat-ended or spherical indenters have been employed in early models of indentation, and the Young's modulus of soft tissues [14–16] can be calculated using the following equation given by Hayes et al. [7]:

(1) $$\begin{array}{c} E=\frac{\left(1-v^{2}\right)}{2ak\left(v,a/h\right)}\cdot \frac{F}{d}, \end{array}$$

where *F* is the indentation force, *v* is the Poisson's ratio of soft tissues, *a* is the radius of indenter, *h* is the tissue thickness, *d* is the indentation depth, and *k* is a scaling factor, which depends on aspect ratio *a*/*h* and Poisson's ratio *v*. It is emphasized that the deformation was assumed to be small, which is difficult to be controlled when indentation test is performed manually [9]. Zhang et al. developed a finite element model using (1) by including the effects of large deformation up to15% strain, and a new set of *k* values were calculated [14].

Young's modulus can be calculated from (1) by using experimentally obtained data of *F* and *d*. The indentation force *F* is normally recorded by a force sensor. The deformation *d* can be measured by optical [17, 18], needle probe [19–21] or ultrasound [9, 22–25] methods. Poisson's ratio *v* is conventionally assumed to be an assigned value [7, 15] or separately measured using other methods [13, 16, 26]. Among them, ultrasound measurement provides a noninvasive and accurate tool for obtaining both tissue thickness and deformation simultaneously. Nevertheless, traditional ultrasound indentation typically operates at the frequency range between 2 and 10 MHz and is normally used to measure the mechanical properties of entire tissue layers. Its resolution is not sufficient to map the mechanical properties of soft tissues with fine structures. Moreover, most of current indentation instruments use a contact way, so that tissue damage caused by the measurement instrumentation cannot be avoided. High frequency ultrasound can improve the resolution to a microscopic level. However, for some technical reason, high frequency ultrasound transducers are not suitable for traditional contact indentation [27]. Considering that ultrasound can propagate through water with very small attenuation, Lu et al. [28] developed a water jet system to achieve noncontact high frequency (20–50 MHz) ultrasound indentation. Water jet not only serves as a soft indenter but also as the coupling medium for high-frequency ultrasound.

The ultrasound water jet indentation system has been employed to obtain modulus image of soft tissues [27], to assess articular cartilage degeneration [29] and to evaluate the bone-tendon junction healing progress [28], which has shown great potential to image the modulus distribution of soft tissues for clinical assessment and diagnosis, and/or to perform indentation tests on small specimens at microscopic levels for biological tissues and other materials. However, all the studies mentioned above measured the stiffness ratio of soft tissue as an indicator of tissue pathological state, which is not the intrinsic property of soft tissue. There's still large discrepancy between the values of stiffness ratio and Young's modulus.

In this study, we simulated the water jet indentation using finite element (FE) analysis to investigate the interaction between fluid (water jet) and solid (soft tissue). With this FE model, the Young's modulus of soft tissue can be calculated using an improved indentation solution based on Hayes' equation by introducing the geometry-, material- and deformation-dependent factor *k*. A new set of scaling factor *k* is presented by considering the finite deformation effect of indentation.

## 2. Methods

**Figure 1:**
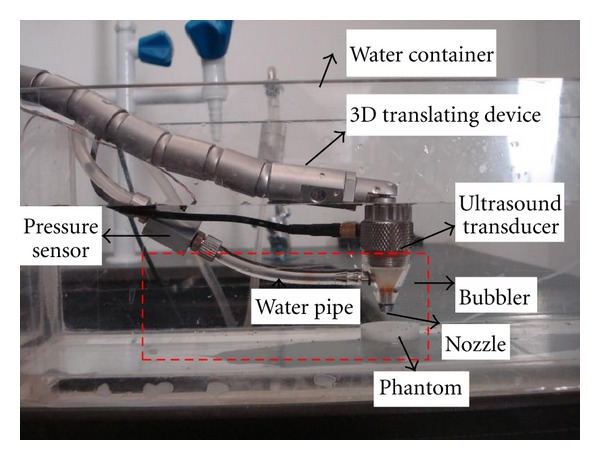
Diagram of the noncontact ultrasound indentation system. The 3D translating device facilitated the system to move easily and monitor the deformation of the phantom. Water flowed from the water pipe and water jet was used as an indenter. Focused high-frequency ultrasound was transmitted through water. The central square region, including a long water pipe, a bubbler, a nozzle, and the soft phantom, was modeled in ANSYS WORKBENCH (see Figure 2). Pressure sensor was located 60 mm from the middle of the bubbler. The dimensions of the important components are: nozzle diameter 1.7 mm, water supply pipe diameter 2 mm, the height of nozzle is 8.5 mm and the distance from the nozzle to tissue approximately 0.95 mm.

The water jet indentation system consists of a 3D translating device (Arthroscopic supporting arm, Medtronic Inc., MN, USA), a water container, a pressure sensor (EPB-C12, Entran Devices, Inc., Fairfield, NJ, USA), a water pipe, an ultrasound transducer (SEUT-506, Acoustic Sensor Co., Ltd., Taiwan), a bubbler, and a nozzle (Figure 1). The 3D translating device facilitates the system to move easily to adjust the distance from the transducer to the tissue sample, and to apply C-scan to obtain modulus image of the region of interest. Focused high-frequency ultrasound is transmitted through the bubbler when it is filled up with water. The central frequency of the ultrasound transducer is 50 MHz, with focal length at 12 mm, and aperture size at 6 mm. The dimensions of the important components are nozzle diameter 1.7 mm, water supply pipe diameter 2 mm, the height of nozzle is 8.5 mm, and the distance from the nozzle to tissue approximately 0.95 mm which is determined by adjusting the ultrasound beam focused at tissue surface. Pressure sensor was located 60 mm from the middle of the bubbler.

**Figure 2:**
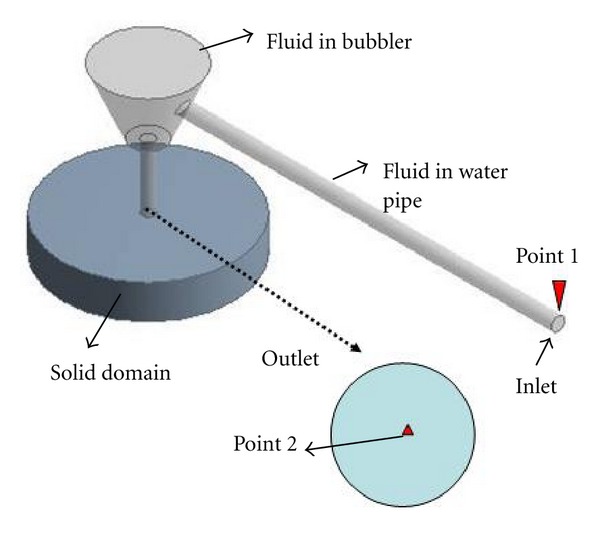
Model of the water jet indentation in ANSYS. The upper part represents fluid domain, which included a long water pipe with 60 mm in length, a bubbler, and a nozzle. To make the outlet clearer, an enlarged part was displayed at the right bottom. Radius of inlet was 1 mm and outlet 0.85 mm. Point 1, the upper point of inlet, and Point 2, the central point of outlet, were set as two monitoring points. The lower part represents solid domain. Diameters of the tissue phantom was 25 mm and its thickness was 5 mm.

A three-dimensional finite element (FE) model whose geometry was as same as the experimental system was established using ANSYS (version 11.0, Canonsburg, PA, USA) to simulate indentation (Figure 2). The interaction between the water jet and soft tissue involves fluid and structural solid coupling, therefore, the simulation was performed by computational fluid dynamics (CFD) analysis that was performed in ANSYS CFX 11 and ANSYS 11 structural codes, coupled through the ANSYS MFX solver. One-way fluid-structure interaction (FSI) theory was applied in this problem.

### 2.1. Model Geometries

The fluid domain model is shown as Figure 2. The dimensions of the important components are totally same as the experimental system described above. The CFX-Mesh method was applied to mesh the fluid domain. With consideration of velocity gradients in near-wall regions, inflation theory was used. In our study, when the flow rate at the inlet was increased from 1 m/s to 10 m/s, different meshes influenced the results significantly. After a proper grid sensitivity analysis, the number of inflated layers was set as 11 and the maximum thickness of inflated boundary was set as 0.1 mm. Triangle elements were generated outside of the boundary layer regions. Refinement of the mesh is implemented at the boundaries. Totally a mesh of 665242 nodes and 2877024 elements were employed for all simulations.

The model of solid part is built up as a cylinder soft tissue with its thickness at 5 mm and diameter at 25 mm. The patch conforming mesher under tetrahedrons is used. Regular meshes consisting of about 101911 nodes and 65034 elements were adopted, after a proper grid sensitivity analysis, for all the models developed.

### 2.2. Boundary Conditions

The simulated indentation test considers a specimen supported by a rigid impermeable plate and indented by water jet from the nozzle. One-way FSI was adopted in our study, therefore, the fluid part and solid part were modeled separately. After fluid models were solved, pressure calculated from fluid outlet was mapped to the contact region of soft tissue.

The following boundary conditions were imposed on all the fluid models. (1) Inlet boundary was adopted at the beginning of the water pipe, with speed varied from 1 m/s to 10 m/s and intensity of 5% of turbulence model was used. (2) Outlet boundary was defined at the interface of fluid and relative pressure was 0 Pa. (3) Wall influence on flow is defined as no slip and wall roughness as smooth wall. As to the solid model, the nodes were constrained in the vertical direction at the bottom of the specimen and pressure was mapped from the fluid outlet to specimen surface.

### 2.3. Material Properties

Water at 22°C was used for the fluid models. Soft tissue was assumed to be a linear-elastic, homogeneous, and isotropic thin layer adhere to a rigid foundation [15, 16]. The mechanical properties were described by Young's modulus *E* and Poisson's ratio *v*. Different values of Young's modulus obtained from literature review and our previous studies, 10 kPa, 52 kPa, 146 kPa, 270 kPa, 740 kPa, and 1000 kPa were assigned to the tissue model in FE analysis to mimic human normal liver, human diseased liver, breast benign lesion, breast malignant lesion, cancerous skin, and articular cartilage, respectively, [30–32]. Density of the soft tissue was 1060 kg/m^3^.

### 2.4. Extract of Young's Modulus of Soft Tissue

Different from the traditional indentation, our water-jet indenter can be regarded as a “soft indenter”, therefore, Hayes' equation cannot be used to derive Young's modulus from the force-displacement curve obtained with our experimental system anymore. An improved indentation solution was proposed by taking into account the finite deformation effect as

(2) $$\begin{array}{c} E=\frac{\left(1-v^{2}\right)}{2ak\left(v,a/h,d/h\right)}\cdot \frac{F}{d}, \end{array}$$

therefore,

(3) $$\begin{array}{c} k\left(v,\frac{a}{h},\frac{d}{h}\right)=\frac{\left(1-v^{2}\right)}{2aE}\cdot \frac{F}{d}, \end{array}$$

where *E* is Young's modulus, *F* is the indentation force which was calculated by the FE simulation, *v* is the Poisson's ratio of soft tissues which was generally assumed as the values reported by literature review, *a* is the radius of indenter and *h* is the tissue thickness which can be obtained in advance before an indentation test, *d* is the indentation depth which was assigned to the nodes on the upper surface of the indenter during the simulation of indentation test, and *k* is a scaling factor, which depends on aspect ratio *a*/*h*, deformation ratio *d*/*h*, and Poisson's ratio *v*.

The simulated force-deformation data were used to calculate the Young's modulus and then derive the *k* value. These new *k* values were calculated using (3). Then the Young's modulus of the soft tissues under water jet indentation can be calculated by substituting these new *k*-values to (2). Different fixed Poisson's ratio (*v* = 0.1–0.5), indentation depth (0.1%–10%), and aspect ratio *a*/*h* (0.17, 0.4, 0.6, 0.8, and 1) were also assigned to the tissue model to investigate the effects of these factors on the estimation of Young's modulus. Therefore, we have a total of 1650 simulation processes in this study.

## 3. Results

**Figure 3:**
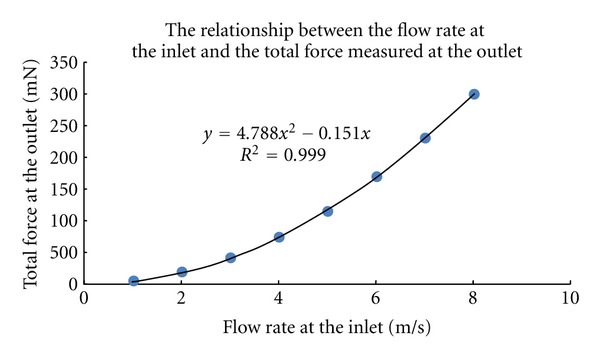
The relationship between the flow rate measured from Point 1 at inlet and the force calculated from Point 2 at outlet.

**Figure 4:**
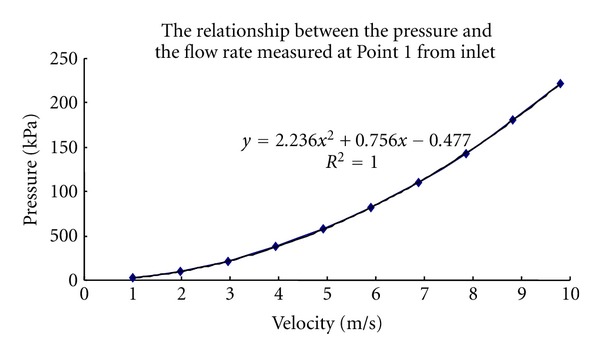
The correlation between the pressure and the flow rate measured both at Point 1 from inlet.

First, the relationship between the pressure (or flow rate) measured at the inlet and the total force applied to the soft tissue was analyzed. This relationship is crucial because it is difficult for us to directly measure the force applied to the soft tissue by water jet during experiment. Two measurement points were set at the inlet and outlet (Points 1 and 2 as indicated in Figure 2), respectively.  Figure 3 shows a quadratic function that fit the flow rate measured from Point 1 at inlet and the force calculated from Point 2 at outlet.  Figure 4 shows the relationship between the flow rate and the pressure measured both at Point 1 at inlet. The nonlinear but monotonic relationship indicates that we may measure only pressure or flow rate at the inlet to calculate the force applied to the soft tissue. It will highly facilitate the water jet indentation when it is used in clinical applications.

Table 1 shows the *k* values with different aspect ratios (0.17–1.0), Poisson's ratios (0.1–0.5), and deformation ratios (0.01–0.1). The *k* values increased with the increase of aspect ratios and Poisson's ratios. However, the nonlinear and non-monotonic relationships between the *k* values and deformation ratio were found.

In comparison with the ground truth, the percentage errors of the calculated Young's moduli are between −0.07% and −1.47% when the aspect ratio varied from 0.17 and 1, which suggested a quite good agreement between the actual and calculated values of Young's modulus.

## 4. Discussion

In this study, a 3D FE model was constructed to simulate the water jet indentation on soft tissues. One-way fluid-solid coupling analysis was conducted to find the relationship among the parameters of the indentation force, tissue deformation, tissue Young's modulus and Poisson's ratio and the aspect parameters, including the indenter size and tissue sample size. An improved Hayes' equation was developed to calculate Young's modulus of soft tissue from the force-deformation curve by introducing a new scaling factor *k*. A new set of *k* values for different Poisson's ratio *v*, aspect ratio *a*/*h*, and strain level *d*/*h* were calculated. We also investigated the relationship of the water parameters, such as pressure and flow rate, in the water jet indentation system and the influence of these parameters on the indentation force. The percentage errors of the estimated Young's modulus ranged from −0.07% to −1.47% in comparison with the assigned values in FE simulation when the aspect ratio *a*/*h* ranged from 0.17 to 1. This robustness of the estimation could be further improved by averaging the parameters calculated using different deformation levels.

It has been demonstrated that our system can measure the Young's modulus of the soft tissue under water jet indentation at one single site. As shown in Figure 1, a flexible arm is used as the supporting arm to control the water jet indenter in this system. This arm, actually utilized the system to conduct C-scan over the tissue sample easily. C-scan imaging provides a useful view of an object, showing a plane perpendicular to the ultrasound beam. It has been widely used in the aerospace industry to detect the surface corrosion, delaminations, voids, cracks and other faults in aging aircraft [33, 34]. In our study, it's easy to apply C-scan with different water pressures for C-scan sequences. After analyzing both the ultrasound signal and flow rate collected from different C-scans, the modulus image could be obtained.

The water jet ultrasound indentation system has been proven that it is useful for the assessment of tissue pathology. Tissue elasticity is one important parameter which generally correlates with tissue pathological changes. This study, for the first time, extracts Young's modulus of soft tissue from water jet indentation using FE analysis. In our FE model, soft tissue is assumed as a linear elastic, homogeneous and isotropic material. However, biphasic theory suggests that most tissues are composed of solid and fluid materials, and they possess very complicated structure-function behaviors and exhibit time-dependent behavior, that is, nonlinear or visco-elastic, heterogeneous and anisotropic behavior. Normally, solid matrix represents the elastic properties while fluid materials represent the viscous properties. In order to interpret these complicated behaviors of soft tissues, the acquired force-deformation data should be interpreted carefully by taking the biphasic theory of soft tissue into accounts.

This study only focused on the nonlinear effect caused by the finite deformation of indentation. The nonlinear and visco-elastic properties of soft tissues have not been addressed yet. Studies on nonlinear tissues' models [35] and indentation of anisotropic biomaterials [36] have been reported. In the future study, the effects of nonlinear visco-elaticity, inhomogeneity and anisotropy should be considered. Another issue is about the structure of the tissue model. In this study, tissue model with a single layer was developed. However, in most cases, soft tissues are multi-layers and behave complex. Our high frequency ultrasound can differentiate tissue layers with high resolution. Hence, for a further study, soft tissues with multi-layers should be considered in the model, so that, both the depth-dependent and site-dependent Young's modulus distribution can be imaged with our system. What's more, experiments about indentation with the water jet indentation system will be conducted, and results will be compared with those obtained from this FE study.

## Figures and Tables

**Table 1:** The scaling factor (*k*) used in this study for different aspect ratios (*a*/*h*), Poisson's ratios (*v*), and deformation ratios (*d*/*h*).

	Poisson's ratio					
*d*/*h *	0.10	0.20	0.30	0.40	0.45	0.50
*a*/*h* = 0.17						
0.001	0.8009	0.8050	0.8112	0.8215	0.8306	0.8379
0.01	0.7924	0.7967	0.8043	0.8171	0.8277	0.8385
0.05	0.7532	0.7565	0.7662	0.7858	0.8015	0.8248
0.07	0.7343	0.7354	0.7471	0.766	0.7825	0.8102

*a*/*h* = 0.4						
0.001	0.9307	0.9422	0.9617	0.9963	1.0254	1.0909
0.01	0.9266	0.9384	0.9589	0.9957	1.0261	1.0961
0.05	0.9074	0.92	0.9437	0.9886	1.0261	1.1129
0.1	0.8827	0.8945	0.9199	0.9712	1.0149	1.1186

*a*/*h* = 0.6						
0.001	1.0721	1.0918	1.1278	1.1948	1.2526	1.3683
0.01	1.0695	1.0896	1.1267	1.1965	1.2564	1.3782
0.05	1.0572	1.079	1.1204	1.2011	1.2715	1.4181
0.1	1.0407	1.0632	1.1087	1.2002	1.2822	1.4643

*a*/*h* = 0.8						
0.001	1.2303	1.2628	1.3208	1.4338	1.5346	1.7026
0.01	1.2289	1.2621	1.3217	1.4368	1.542	1.7191
0.05	1.2225	1.2581	1.3240	1.4566	1.5771	1.7923
0.1	1.2135	1.2513	1.3237	1.4744	1.6152	1.8761

*a*/*h* = 1.0						
0.001	1.4239	1.4715	1.5601	1.7385	1.9	2.197
0.01	1.4238	1.4722	1.5628	1.7465	1.9128	2.2257
0.05	1.4227	1.4746	1.5738	1.7803	1.973	2.3573
0.1	1.4204	1.4759	1.5847	1.8364	—	—
